# Training the trainers in emergency medicine: an advanced trauma training course in Rwanda's medical simulation center

**DOI:** 10.11604/pamj.2015.20.242.6358

**Published:** 2015-03-13

**Authors:** Hannah Janeway, Payal Modi, Grace Wanjiku, Ramon Millan, Devin Kato, John Foggle, Robert Partridge

**Affiliations:** 1 The Warren Alpert Medical School of Brown University, Rwanda; 2 Department of Emergency Medicine, The Warren Alpert Medical School of Brown University, Rwanda

**Keywords:** Trauma, emergency medicine, Rwanda, low-cost simulation, international

## Brief

Emergency medicine (EM) has undergone rapid development in Rwanda and the delivery of healthcare has steadily improved over the past two decades [1]. Life expectancy has risen while maternal and child mortality have declined [2]. However, trauma remains a leading cause of morbidity and mortality in Rwanda, similar to other countries in Sub-Saharan Africa [3–5]. Therefore, the Human Resources for Health (HRH) and the Rwanda Ministry of Health have made acute EM and trauma training a priority to improve patient care and public health outcomes [6]. Faculty from the Rwandan Post Graduate Degree (PGD) Course in Emergency and Critical Care Medicine and the Masters of Medicine in Emergency Medicine, both based at the University Teaching Hospital of Kigali (CHUK), have collaborated with providers from two US-based Medical Schools and Emergency Department to help improve EM in Rwanda. In October 2013, a group of eight EM providers were invited to Rwanda to conduct an intensive course in emergency trauma care, with the focus on providing care in resource-limited settings. The course, originally developed for Nicaragua, was specifically redesigned to meet the needs and resources in Rwanda. Previous results showed significant improvement in knowledge through simulation and written assessments [7]. The course was conducted at the Centre for Simulation and Training at CHUK, Rwanda's new and only facility for medical simulation in conjunction with CHUK faculty from the surgery and EM deparments [8]. The 3-day trauma course was developed to address major concepts in trauma care, while accounting for local injury patterns and resource constraints in Rwanda. Training consisted of didactic lectures intermixed with two procedure labs, four live simulation cases, and team-building workshops. Fourteen Rwandan residents participated of which eleven were in the Rwandan PGD course in Emergency and Critical Care Medicine, two were from Family Medicine, and one was from Internal Medicine.

**Core Content:** participants received didactic lectures from US and Rwanda based EM and surgical faculty. The 14 lectures covered basic trauma concepts including shock, head trauma and spinal injuries as shown in Table 1. Previously recorded video demonstrations were utilized to demonstrate the primary/secondary survey, effective communication, and teamwork. Trainees then participated in interactive simulation activities involving residents and CHUK nurses to practice learned skills during a trauma resuscitation and to receive real-time feedback from colleagues and course faculty.

**Table 1:** Course content

Lectures	Simulation Cases	Procedure labs/Interactive Sessions
Intro/Trauma Overview	Closed Head Injury (1)	Thoracostomy
CHUK Triage	Blunt Traum in Pregnancy (2)	Ultrasound: FAST Exam
Primary/Secondary Survey	Gun Shot Wound to Chest (3)	Primary/Secondary Survey Lab
Shock	MCC with Splenic Laceration (4)	Teambuilding Workshop
Chest Trauma		
**Abdominal/Pelvic Trauma**		
Thoracostomy/FAST		
Head Trauma		
Cervical Spine Trauma		
Teamwork in Trauma		
Extremity Trauma		
Pediatric Trauma		
Elderly Trauma		
Trauma in Pregnancy		

**Procedure Labs:** procedure stations were designed in collaboration with residency directors to address skill deficits of the residents. Didactic sessions were conducted covering proper technique and methodology. Participants were then divided into smaller groups and rotated through two procedure stations: tube thoracostomy and focused assessment sonography for trauma (FAST). Simulation equipment was designed using low cost materials including rehabilitated CPR manniquins and locally available equipment so that participants could replicate this teaching technique with providers at their home institutions.

**Figure 1:**
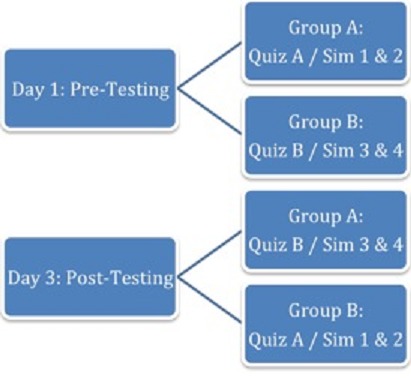
Pre and post-course testing and assessment

**Practicum:** on days 1 and 3, participants were randomly assigned to Group A or Group B and underwent pre- and post-training evaluations consisting of a 23-question written exam (Test A or B) and 2 distinct simulation cases (cases 1 and 2 or cases 3 and 4), (See Figure 1). All forty-six written questions were reviewed for content by US board certified physicians in EM and surgery.

**Simulation Cases:** medical simulation cases teaching trauma assessment and management were modified for the Rwandan context. Simulations were performed utilizing course faculty as actors in roles as patient, nurse and case facilitator (e.g. adjusted simulated patient's clinical condition based on management by course trainees). Simulation cases received two scores: 1) a completion checklist scored out of 100 and 2) time elapsed to complete pre-identified actions. Themes for the four simulation cases are shown in Table 1. Completion check-lists and critical actions were tailored for each simulation case. Simulations were video-taped for residency development purposes. A debriefing session at the end of the course provided feedback to participants regarding management strengths and pitfalls. All 14 participants completed the didactic and simulation components of the course, and performance was formally assessed with feedback to participants. A follow-up trauma skills and referesher course is planned to ensure knowledge and practice retention. In addition, CHUK EM faculty are regularly monitoring the Rwandan physicians when they are managing trauma patients to ensure that the skills taught in the course are utilized. The medical simulation and skills center was an effective venue for the delivery of intensive trauma training. Since the course was designed with a “train the trainer” approach, it is anticipated that the medical simulation and skills center will continue to demonstrate its value as future clinicians in-training are educated in this center exculsively by Rwandan faculty.

## References

[1] Kabeza Antoine, George N, Nyundo M, Levine AC (2013). Development of Emergency Medicine in Rwanda. African Journal of Emergency Medicine..

[2] Progress Rwanda Health Indicators June 2012 http://moh.gov.rw/english/?page_id=2212.

[3] Nsereko Etienne, Brysiewicz P (2010). Injury surveillance in a central hospital in Kigali, Rwanda. J Emerg Nurs.

[4] Twagirayezu E, Teteli R, Bonane A, Rugwizangoga E (2008). Road traffic injuries at Kigali University Central Teaching Hospital, Rwanda. East and Central African Journal of Surgery..

[5] Ademuyiwa Adesoji O, Usang UE, Oluwadiya KS, Ogunlana DI, Glover-Addy H, Bode CO, Arjan BV (2012). Pediatric trauma in sub-Saharan Africa: Challenges in overcoming the scourge. J Emerg Trauma Shock..

[6] Henwood Patricia C, Rempell JS, Liteplo AS, Murray AF (2013). Point-of-care ultrasound use over six-month training period in Rwandan district hospitals. African Journal of Emergency Medicine..

[7] Pringle K, Mackey J, Ruskis J, Modi P, Foggle J, Levine AC (2013). A short trauma course for physicians in a resource-limited setting: is low-cost simulation effective?. Ann Emerg Med..

[8] Livingston Patricia, Bailey J, Ntakiyiruta G, Mukwesi C, Whynot S, Brindley P (2014). Development of a simulation and skills centre in East Africa: a Rwandan-Canadian partnership. Pan African Medical Journal..

